# Biomechanical Comparative Evaluation of the Atrophic Edentulous Mandible Using All-on-4, All-on-5 and All-on-6 Concepts: A Finite Element Analysis

**DOI:** 10.3390/dj14080480

**Published:** 2026-08-05

**Authors:** Abhay Datarkar, Neha Ganpat Pawar, Prashant K. Pandilwar, Ihsan Caglar Cinar, Eitan Mijiritsky

**Affiliations:** 1 Department of Oral and Maxillofacial Surgery, Government Dental College and Hospital Nagpur, Nagpur 440003, Maharashtra, India; abhaydatarkar@yahoo.com (A.D.); drneha7758@gmail.com (N.G.P.); prashant.pandilwar@rediffmail.com (P.K.P.); 2 Department of Oral Implantology, Faculty of Dentistry, Istanbul University, Fatih, Istanbul 34098, Turkey; cinarcaglar@gmail.com; 3 Department of Head and Neck Surgery and Maxillofacial Surgery, Tel-Aviv Sourasky Medical Center, School of Dental Medicine, Gray Faculty of Medical and Health Sciences, Tel Aviv University, Tel Aviv 64239, Israel

**Keywords:** finite element analysis, atrophic mandible, dental implant, biomechanical evaluation, stress distribution

## Abstract

**Background**: Full-arch implant-supported prostheses, including configurations such as All-on-4, All-on-5, and All-on-6, are widely used for the rehabilitation of the atrophic edentulous mandible; however, their biomechanical performance remains a critical factor for long-term success. **Aim/Objectives**: To evaluate and compare von Mises stress distribution and deformation patterns among mandibular All-on-4, All-on-5, and All-on-6 configurations using three-dimensional finite element analysis. **Methods**: The three-dimensional finite element models of an atrophic edentulous mandible were developed to simulate All-on-4 (Model 1), All-on-5 (Model 2), and All-on-6 (Model 3) implant-supported prostheses under standardized loading conditions (150 N anterior; 300 N posterior bilaterally). Von Mises stress (overall, implant, and bone) and total deformation were assessed. The results were analyzed using descriptive statistics, and a comparative evaluation of von Mises stress and deformation values was performed across the three implant configurations. **Results**: Model 2 exhibited the highest overall von Mises stress (76.821 MPa), followed by Model 3 (67.996 MPa) and Model 1 (38.016 MPa). Bone stress was lowest in Model 1 (5.787 MPa) and highest in Model 2 (16.443 MPa). Total deformation was greatest in Model 2 (3.156 × 10^−3^ mm), whereas Model 3 showed the least deformation (8.2489 × 10^−4^ mm). **Conclusions**: Within the limitations of this study, the All-on-5 configuration demonstrated the highest stress concentration and deformation under the present loading conditions. Increasing implant number does not linearly reduce stress. Among the three evaluated configurations, the modeled All-on-6 configuration demonstrated the lowest deformation under the present loading conditions. Implant positioning and load distribution play a more critical role than implant number alone. The findings are based on a specific finite element model and implant distribution and should not be generalized to all clinical All-on-5 configurations.

## 1. Introduction

Edentulism of the mandible continues to represent a significant clinical challenge, particularly in elderly populations, and is frequently associated with progressive alveolar bone resorption. This resorptive process leads to a reduction in bone volume and density, resulting in compromised prosthetic support, decreased masticatory efficiency, altered phonetics, and a substantial decline in patient quality of life. In cases of advanced mandibular atrophy, conventional complete dentures often fail to achieve adequate retention and stability due to unfavorable ridge morphology and reduced denture-bearing surface area. Consequently, implant-supported rehabilitation has become the standard of care, offering predictable functional outcomes and improved patient satisfaction compared with removable prostheses [1,2].

Implant-supported full-arch prostheses have transformed the management of edentulous mandibles by restoring occlusal function, enhancing prosthetic stability, and improving overall oral health–related quality of life. Among the available treatment strategies, the All-on-4 concept has gained widespread clinical acceptance as a minimally invasive, graftless solution for rehabilitating severely resorbed jaws. This approach involves the placement of two anterior axial implants in the interforaminal region and two posterior implants tilted distally, thereby maximizing the utilization of available bone, minimizing cantilever length, and avoiding critical anatomical structures [3]. Long-term clinical studies have demonstrated high survival rates and favorable outcomes with this concept. However, biomechanical concerns persist, particularly regarding stress concentration in distal implants and peri-implant bone under functional loading conditions, which may contribute to marginal bone loss and mechanical complications over time [4,5].

In an effort to enhance biomechanical performance and optimize load distribution, alternative configurations such as All-on-5 and All-on-6 have been proposed. Increasing the number of implants is theoretically expected to improve load sharing, reduce stress concentration, and enhance the rigidity of the prosthetic framework. However, current evidence remains inconclusive, with several recent investigations suggesting that implant number alone may not be the primary determinant of biomechanical success. Instead, factors such as implant positioning, angulation, anterior–posterior spread, and prosthetic design appear to play a more critical role in governing stress distribution patterns and overall system stability [6].

These findings underscore the need for a comprehensive biomechanical evaluation of different implant configurations, particularly in anatomically compromised conditions such as the atrophic mandible. Although several finite element studies have compared All-on-4 and All-on-6 configurations, the intermediate All-on-5 configuration has received relatively limited attention in the literature. Clinically, All-on-5 may be considered in situations where additional implant support is desired without significantly increasing surgical complexity or cost associated with six implants. However, its biomechanical effectiveness remains unclear, particularly whether the addition of a fifth implant provides a meaningful improvement in load distribution or represents a transitional configuration without substantial biomechanical advantage. Therefore, evaluating the biomechanical behavior of the All-on-5 configuration is essential to determine its clinical relevance and to address this gap in the existing literature [7,8].

Finite element analysis (FEA) has emerged as a powerful and widely accepted computational tool for investigating biomechanical behavior in implant dentistry [9]. As a non-invasive and highly versatile method, FEA enables detailed simulation of stress distribution, strain patterns, and deformation within complex anatomical structures under controlled loading conditions [10]. It allows precise evaluation of implant–bone interactions and facilitates comparative analysis of different implant configurations without the ethical and practical constraints associated with clinical studies [11]. Furthermore, FEA is particularly advantageous in analyzing atrophic mandibles, where variations in bone quality and geometry can significantly influence biomechanical outcomes [12].

Despite the growing body of literature, there remains a lack of consensus regarding the optimal number of implants for full-arch mandibular rehabilitation [13]. Many existing studies have either focused on limited configurations or have not comprehensively evaluated both stress distribution and deformation characteristics under standardized conditions. Moreover, the interplay between implant number, load transfer mechanisms, and structural rigidity in atrophic mandibles is not yet fully understood [14,15,16].

Therefore, the aim of the present study was to perform a detailed three-dimensional finite element analysis to evaluate and compare the biomechanical performance of three specific All-on-4, All-on-5, and All-on-6 implant configurations in an atrophic edentulous mandible. Particular emphasis was placed on assessing von Mises stress distribution and total deformation within the implant system and surrounding bone under simulated occlusal loading conditions, with the objective of identifying biomechanically favorable configurations that may inform clinical decision-making.

The present study compares three specific implant configurations representing All-on-4, All-on-5, and All-on-6 treatment concepts. Because implant number and implant spatial distribution differ simultaneously among these models, the study evaluates the biomechanical behaviour of specific modeled configurations rather than implant number as an isolated variable.

The novelty of this study lies in the comparative evaluation of specific implant distribution models under controlled finite element conditions, rather than proposing a universally applicable clinical protocol.

## 2. Materials and Methods

### 2.1. Study Design and Setting

This study was designed as a three-dimensional finite element analysis (FEA) to evaluate the biomechanical behavior of different implant-supported full-arch configurations in an atrophic edentulous mandible. The study was conducted in the Department of Oral and Maxillofacial Surgery at our institution (Government Dental College and Hospital, Nagpur).

### 2.2. Ethical Approval

The study was approved by the Institutional Ethics Committee of Government Dental College and Hospital, Nagpur (Approval No.: IEC/GDCHN/35/2025; Date: 27 January 2025). Written informed consent was obtained from the patient prior to data acquisition.

### 2.3. Case Selection and Data Acquisition

A three-dimensional finite element model of the mandible was developed using computed tomography (CT) (Siemens Healthineers, Forchheim, Germany) data obtained from a 50-year-old female patient presenting with a moderately atrophic edentulous mandible. The term “moderately atrophic mandible” was defined according to the Cawood and Howell classification, corresponding to Class IV, which is characterized by a knife-edge alveolar ridge with reduced buccolingual width and moderate residual height [17].

CT imaging was performed using a high-resolution multi-slice CT scanner under the following parameters:

• Slice thickness: 1 mm;
• Field of view: covering entire mandible;
• Voxel size: non-uniform (as per scanner settings).

The acquired DICOM data were anonymized prior to processing. The selected mandible exhibited reduced alveolar height and width consistent with moderate atrophy, as described in previously established anatomical classifications.

### 2.4. Image Processing and Geometric Model Reconstruction

The CT data in Digital Imaging and Communications in Medicine (DICOM) format were imported into BlueSky Bio software (Version 4.12, Blue Sky Bio LLC, Libertyville, IL, USA) for segmentation of the mandibular bone.

Segmentation was performed using threshold-based and manual refinement techniques to accurately delineate cortical and cancellous bone structures. The segmented model was converted into stereolithography (STL) format using surface triangulation.

The STL model was further processed to eliminate artifacts, smooth surface irregularities, and achieve a geometrically consistent representation while preserving essential anatomical features such as cortical boundaries, alveolar ridge contours, and mental foramen regions.

Implant planning and positioning were carried out using Geometric Freeform (Version 2025, Oqton Inc., Rock Hill, SC, USA), allowing precise control over implant angulation, spacing, and placement relative to anatomical landmarks.

### 2.5. Finite Element Model Construction

The finalized STL model was imported into ANSYS Workbench (Version 2022 R1, ANSYS Inc., Canonsburg, PA, USA) for finite element modeling and numerical analysis. The mandible was modeled as a two-phase structure consisting of a 2 mm thick cortical bone layer surrounding an inner cancellous core.

All materials were assumed to be homogeneous, isotropic, and linearly elastic, which is a standard simplification in finite element simulations [18]. Material properties assigned to each component were derived from previously published literature and are summarized in Table 1.

### 2.6. Implant Design and Configuration

**Figure 1:**
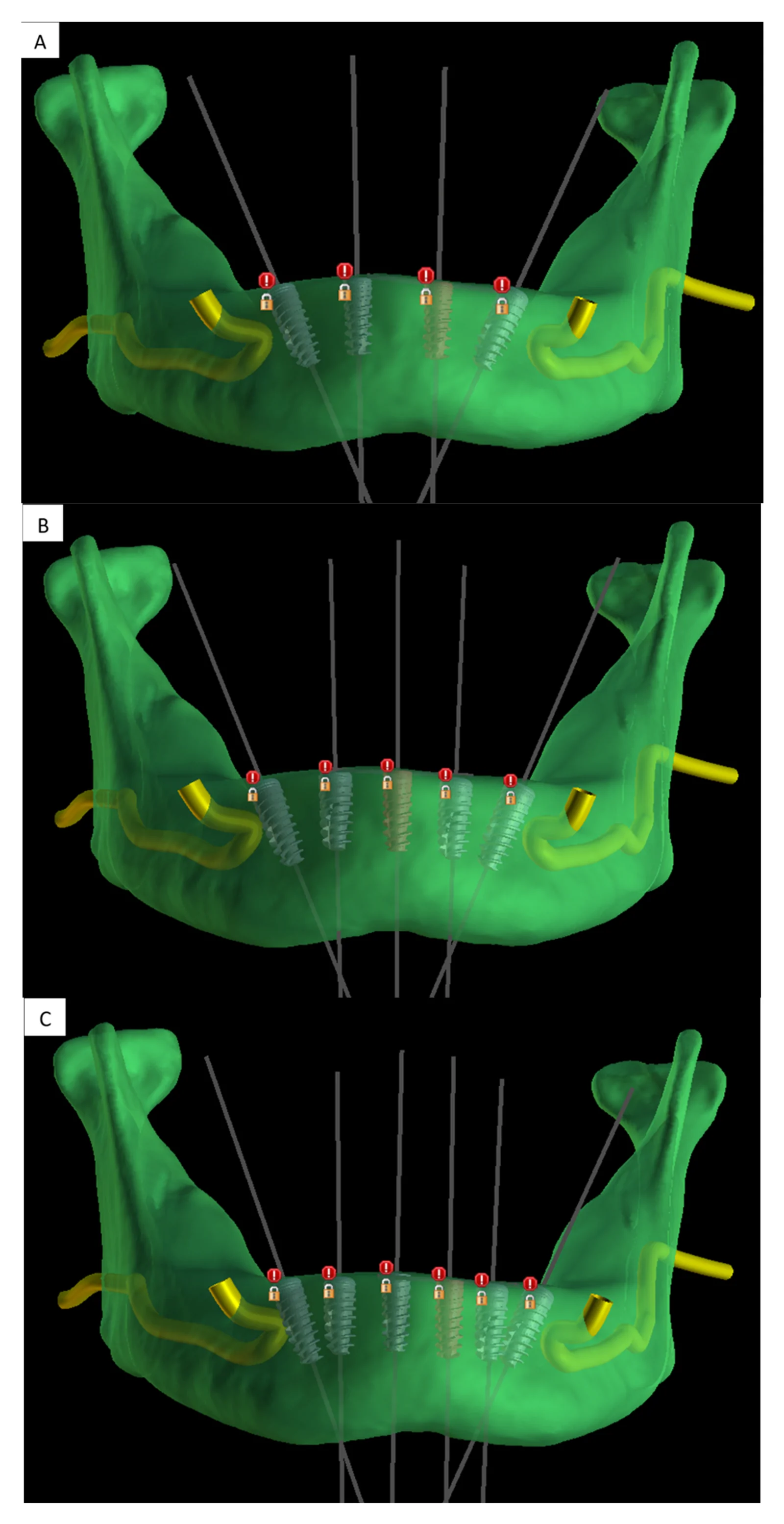
Implant configuration (**A**) Model 1 (All-on-4): two anterior axial implants (at lateral incisor region) placed in the interforaminal region and two posterior tilted implants; (**B**) Model 2 (All-on-5): three anterior axial implants (one at central incisor region and two at canine region) and two posterior tilted implants; (**C**) Model 3 (All-on-6): four anterior axial implants (two at central incisor region and two at canine region) and two posterior tilted implants.

Three different implant configurations were modeled: Model 1 (All-on-4), Model 2 (All-on-5), and Model 3 (All-on-6). All implants were modeled as threaded, root-form endosseous implants (Figure 1). The geometric model was constructed using implant dimensions obtained from the manufacturer’s catalogue (Touareg™ implant system, Adin Dental Implant Systems Ltd., Afula, Israel), ensuring accurate representation of implant morphology.

The implant dimensions were as follows:

• Posterior tilted implants (30° angulation): 4.2 × 13 mm;
• Anterior axial implants (central/lateral incisor region): 3.5 × 10 mm;
• Anterior axial implants (canine region): 3.75 × 10 mm.

The implantation depth was defined relative to the alveolar crest, with all implants placed at the crestal bone level and the implant platform flush with the cortical bone. The anterior implants were inserted to a depth of 10 mm, while the posterior tilted implants were inserted to a depth of 13 mm along their long axis. The coronal portion of the implants engaged the cortical bone, while the apical portion extended into the cancellous bone to simulate clinical conditions.

Implants were positioned anterior to the mental foramen in accordance with standard All-on-X protocols. Anterior implants were placed axially while posterior implants were inserted at 30° angulation. A minimum inter-implant distance of ≥3 mm was maintained between adjacent implants in all configurations, to ensure symmetrical distribution and biomechanical consistency.

The implant distributions were selected to represent clinically relevant treatment concepts while respecting anatomical constraints of the atrophic mandible. Since implant number and spatial distribution were modified simultaneously, comparisons among the models should be interpreted as comparisons of specific implant configurations rather than implant number alone.

A fully bonded contact condition was assumed at the bone–implant interface to simulate complete osseointegration. All implant–prosthesis interfaces were also defined as bonded.

A rigid full-arch splinted prosthetic framework fabricated from titanium alloy (Ti-6Al-4V) was modeled connecting all implants in each configuration. The framework incorporated a 10 mm distal cantilever bilaterally, which was kept identical across all three models. The mechanical properties of the framework were assigned according to those of titanium alloy (Table 1). The framework material, geometry, cross-sectional dimensions, distal cantilever length, and loading points were standardized and kept identical across all three models to ensure that implant configuration was the only variable influencing the biomechanical response.

### 2.7. Mesh Generation and Convergence Analysis

The finite element model was discretized using higher-order tetrahedral elements. A non-uniform meshing strategy was employed, with localized refinement in regions of interest, particularly at the implant–bone interface, to accurately capture stress gradients and complex geometric features. Curvature-based refinement and proximity controls were activated to ensure adequate resolution of intricate anatomical contours, resulting in element sizes as small as approximately 0.08 mm in critical regions.

A mesh convergence analysis was performed using a systematic successive refinement approach, wherein multiple mesh densities were generated and compared. The complete finite element mesh is shown in Supplementary Materials. The convergence criterion was based on the stabilization of peak von Mises stress values at critical regions, specifically the crestal bone surrounding the implant neck, which represents a primary stress concentration zone in implant biomechanics. The variation in peak von Mises stress between successive mesh refinements was found to be less than 5%, indicating mesh-independent results and acceptable numerical accuracy. This approach is consistent with established finite element methodologies based on convergence of key response variables with mesh refinement [19]. A detailed convergence table has not been included because the objective was numerical verification rather than comparison of mesh densities.

The final optimized mesh consisted of approximately 1,278,852 nodes and 902,717 elements. The initial default element size was program-controlled (approximately 8.7 mm), with significant local refinement applied through curvature and geometric controls, ensuring accurate stress distribution while maintaining computational efficiency.

### 2.8. Loading and Boundary Conditions

Static occlusal loading was applied to simulate physiological masticatory forces [20]. A vertical load of 150 N was applied to the anterior (incisal) region and 300 N bilaterally to the posterior (premolar–molar) regions via defined nodes on the prosthetic framework.

All forces were directed axially, perpendicular to the occlusal plane (−Z axis), and distributed across multiple nodes to simulate physiological load transfer to the implants. No lateral or oblique forces were considered.

Boundary conditions were defined by fixing all degrees of freedom at the inferior border of the mandible to prevent rigid body motion; the condylar regions were not modeled.

The ANSYS Workbench solver (ANSYS WORKBENCH 2022R1, ANSYS Inc., Canonsburg, PA, USA) computed stress distribution, displacement, and deformation for each node and element within the model.

### 2.9. Outcome Measures and Analysis

Following load application, the simulation computed total structural deformation (mm) and von Mises (VM) stress (MPa) distribution in the implants and surrounding bone were recorded for each implant configuration.

The VM stress was used as a comparative parameter to evaluate stress distribution patterns across different implant configurations. Implant configurations exhibiting lower stress concentration and more uniform stress distribution were considered biomechanically favorable.

As this study is based on a deterministic finite element model, no conventional inferential statistical analysis was performed. The computed results represent exact numerical outputs under predefined material properties and boundary conditions. Comparative evaluation among the different implant configurations was therefore conducted using descriptive analysis of peak von Mises stress and total deformation values.

## 3. Results

A quantitative and comparative evaluation of von Mises stress distribution and deformation patterns across the three implant configurations (All-on-4, All-on-5, and All-on-6) is presented in Table 2. The results demonstrate distinct biomechanical trends influenced by implant number and spatial distribution.

### 3.1. Overall Von Mises Stress Distribution

**Figure 2:**
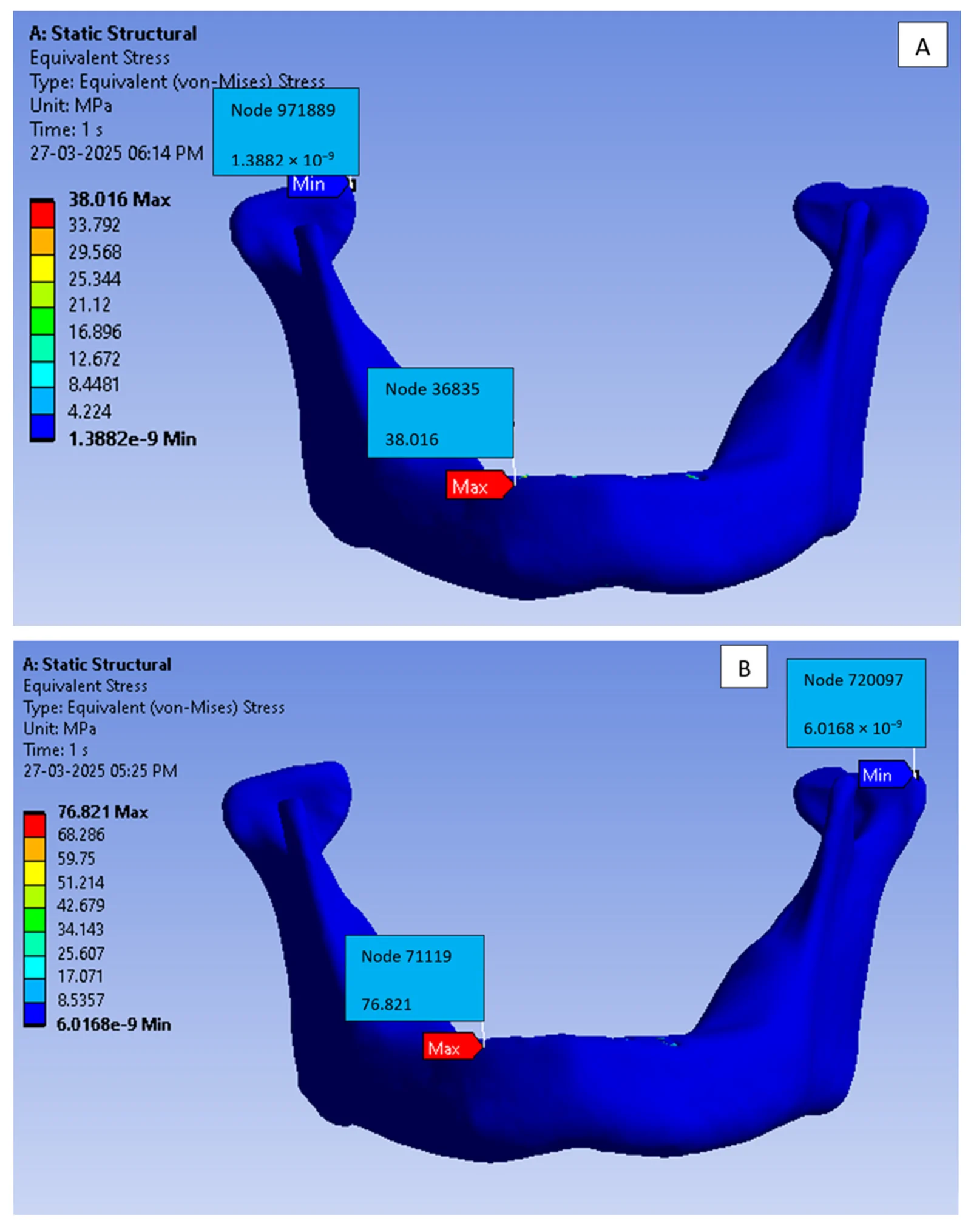

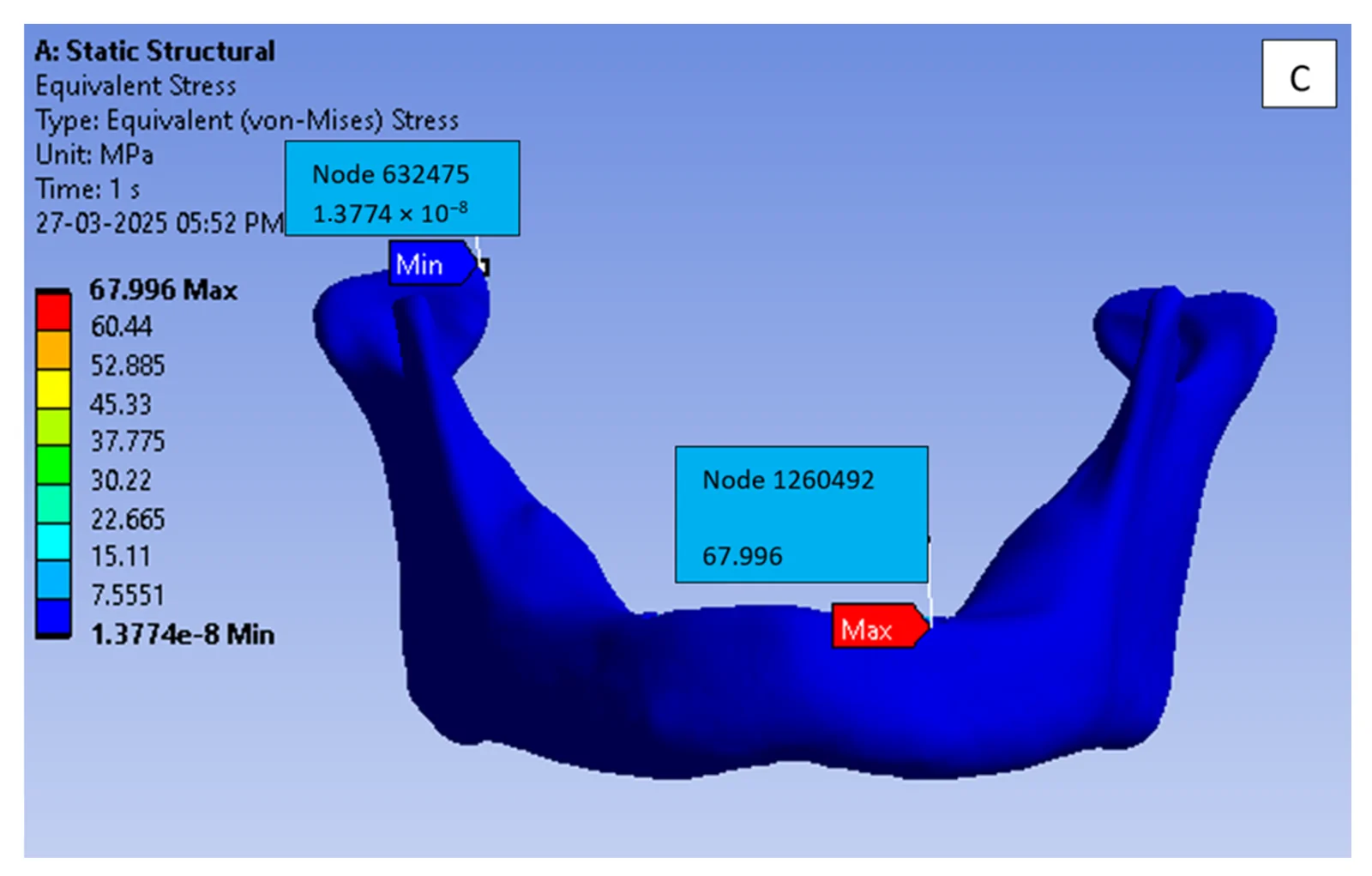
(**A**) Overall stress in model 1; (**B**) overall stress in model 2; (**C**) overall stress in model 3.

The overall von Mises stress demonstrated a non-linear relationship with increasing implant number. The stress increased from 38.016 MPa in Model 1 (All-on-4) to a peak of 76.821 MPa in Model 2 (All-on-5), followed by a reduction to 67.996 MPa in Model 3 (All-on-6). These findings indicate that the All-on-5 configuration produced the highest stress concentration, while increasing the number of implants from five to six improved load distribution, although not to levels observed in the All-on-4 model. Stress distribution was predominantly localized around distal implants in the posterior regions subjected to higher occlusal loads (Figure 2).

This trend may be attributed to altered load transfer in the All-on-5 configuration, where the additional implant does not achieve symmetrical force distribution, leading to stress concentration. In contrast, the All-on-6 configuration provides more balanced load sharing.

### 3.2. Implant-Level Stress Analysis

**Figure 3:**
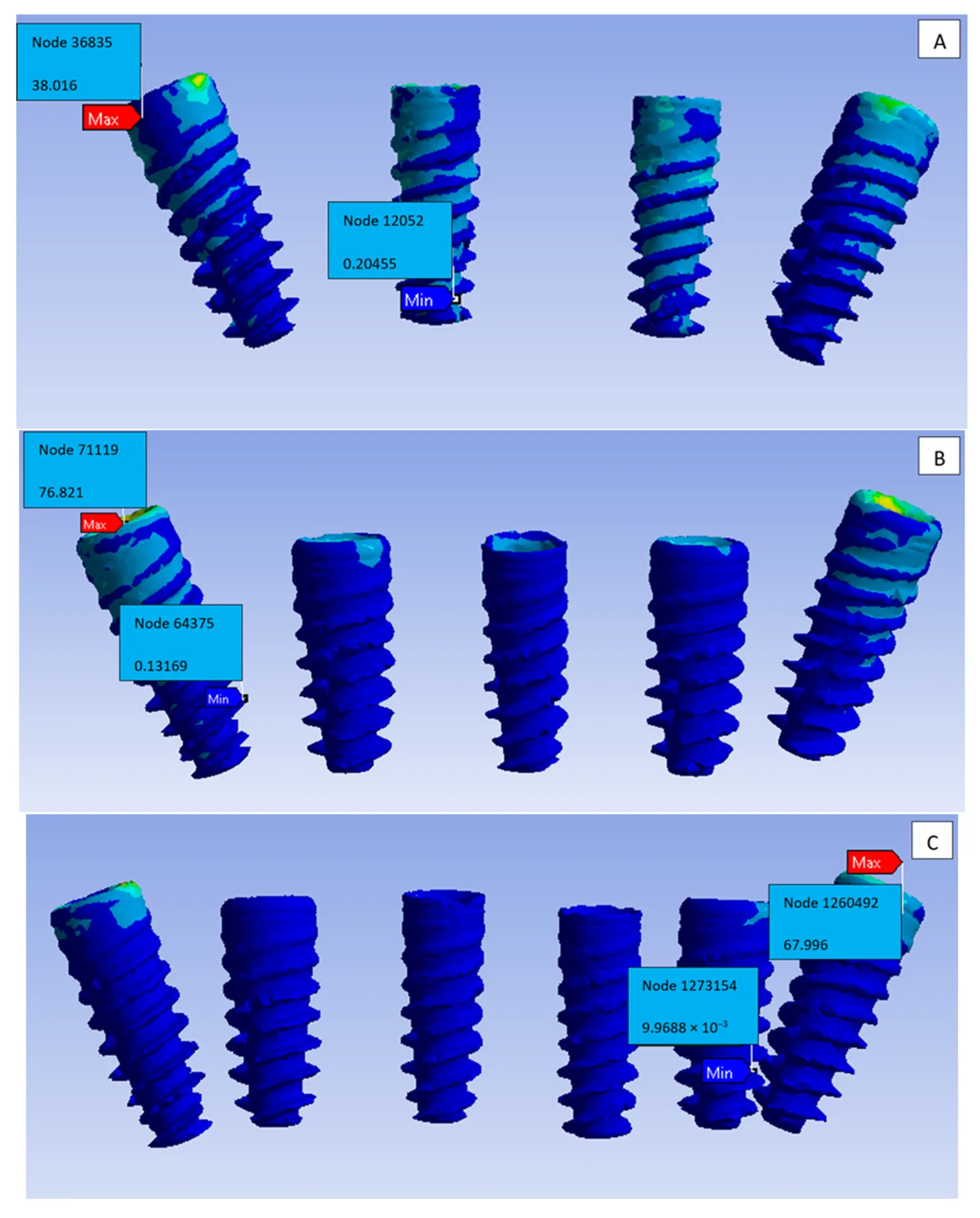
(**A**) Implant stress distribution in model 1; (**B**) implant stress distribution in model 2; (**C**) implant stress distribution in model 3.

A similar trend was observed in implant-level stress. Model 2 exhibited the highest implant stress (76.821 MPa), followed by Model 3 (67.996 MPa) and Model 1 (38.016 MPa). The All-on-5 configuration demonstrated inefficient load sharing, leading to stress amplification, whereas the All-on-6 configuration distributed forces more effectively across multiple implants, thereby reducing peak stress per implant. Stress concentration was primarily observed at the implant neck region and at the implant–bone interface, particularly in posterior tilted implants (Figure 3). The higher stress in the All-on-5 model may result from uneven load sharing and suboptimal inter-implant spacing, causing localized stress concentration, particularly in posterior implants.

### 3.3. Bone Stress Distribution

**Figure 4:**
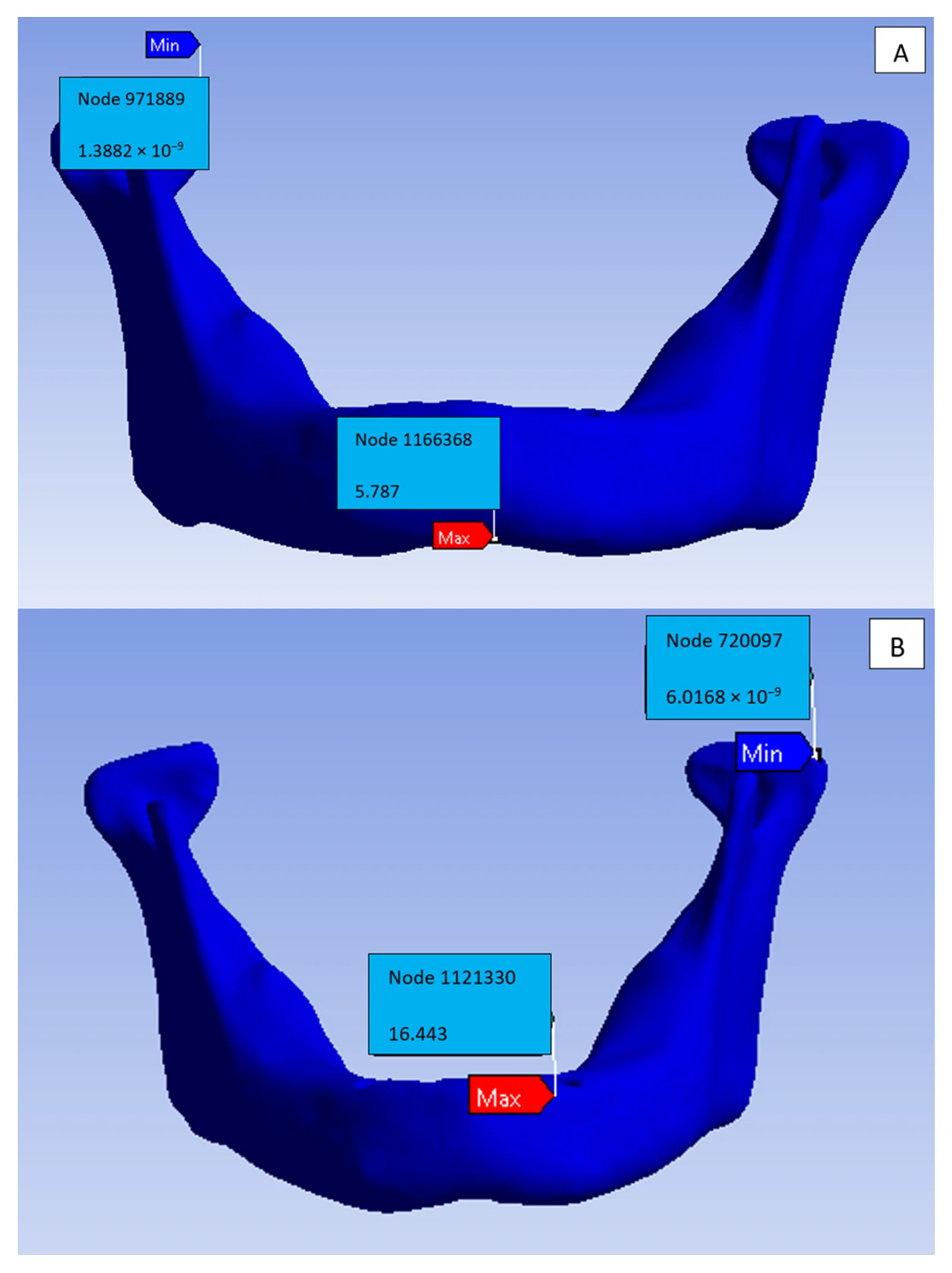

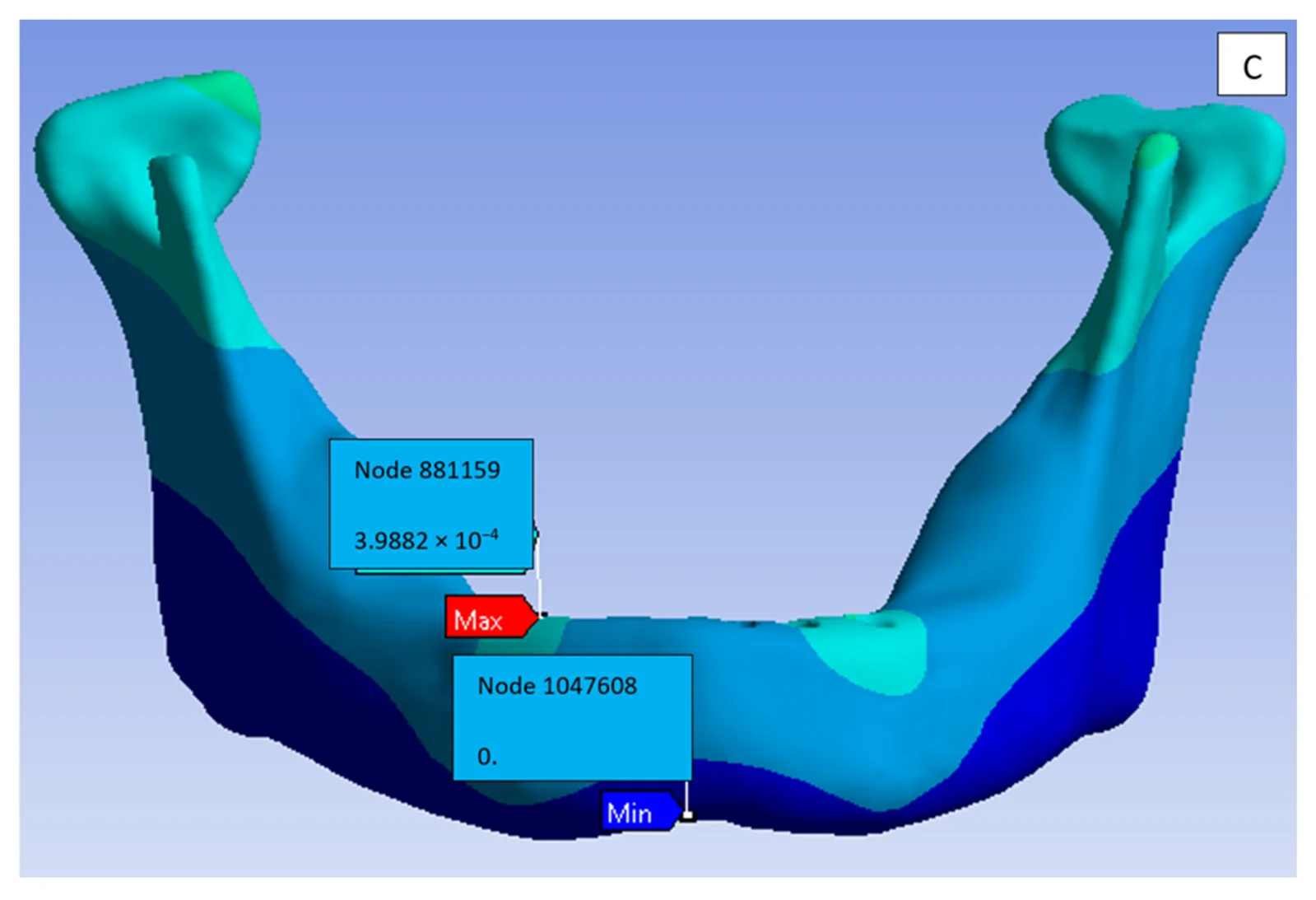
(**A**) Bone stress distribution in model 1; (**B**) bone stress distribution in model 2; (**C**) bone stress distribution in model 3.

Peri-implant bone stress followed a different trend. The lowest bone stress was observed in Model 1 (5.787 MPa), while higher values were recorded in Model 2 (16.443 MPa) and Model 3 (13.486 MPa). This pattern indicates a marked increase from Model 1 to Model 2, followed by a moderate reduction in Model 3. These findings suggest that increasing implant number does not necessarily reduce bone stress; rather, load transfer to the surrounding bone depends on implant configuration and distribution. Stress concentration was mainly observed in the cortical bone surrounding the implant neck region, with minimal stress in cancellous bone (Figure 4).

### 3.4. Total Deformation Analysis

**Figure 5:**
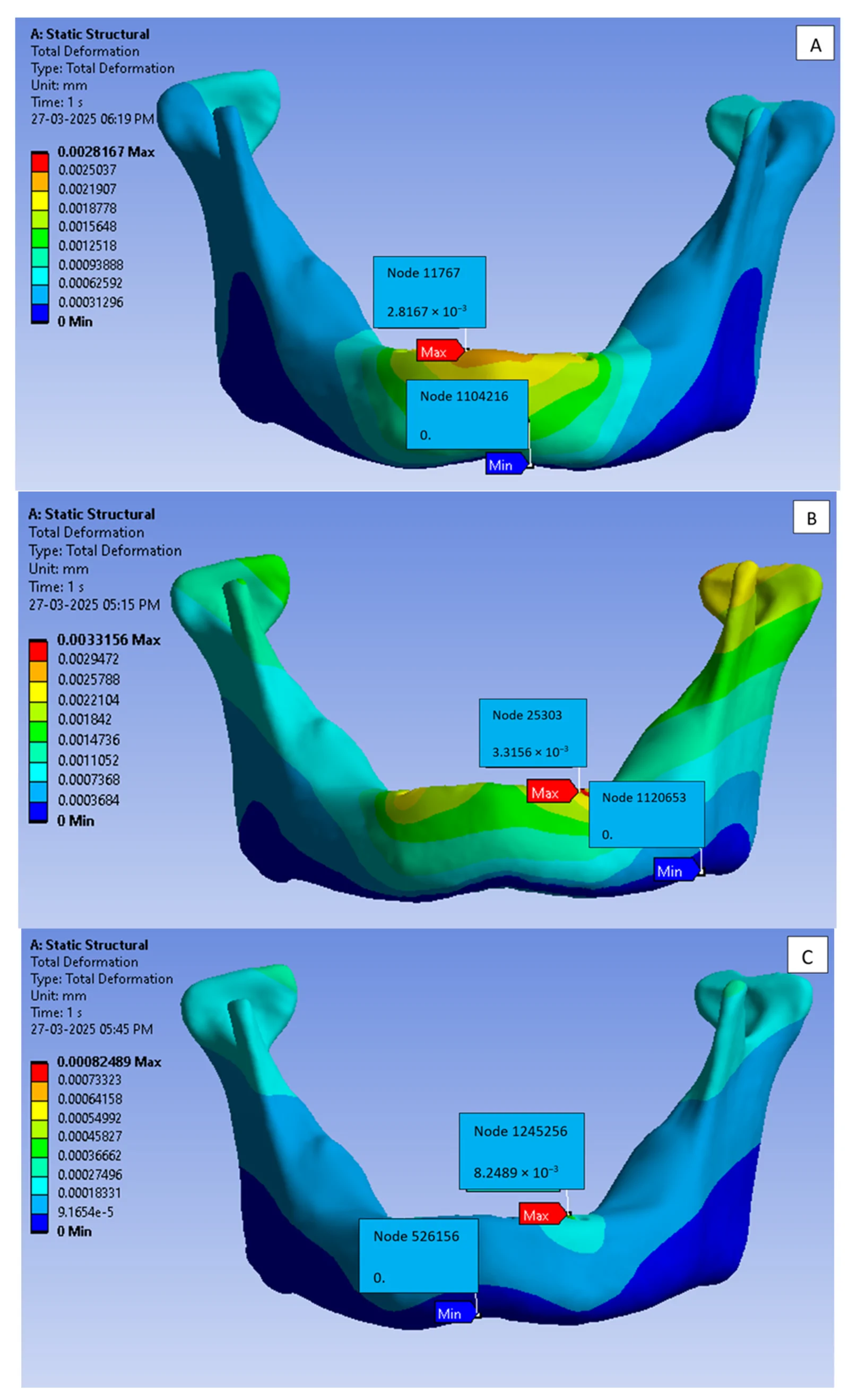
(**A**) Overall deformation in model 1; (**B**) overall deformation in model 2; (**C**) overall deformation in model 3.

Total deformation demonstrated a non-linear trend across the three configurations, with values of 2.8167 × 10^−3^ mm for Model 1, increasing to a maximum of 3.156 × 10^−3^ mm in Model 2, and subsequently decreasing markedly to 8.2489 × 10^−4^ mm in Model 3. This pattern indicates a peak deformation in Model 2 followed by a marked reduction in Model 3, reflecting lower deformation and improved structural rigidity with the addition of a sixth implant. These findings indicate that, among the three evaluated configurations, the modeled All-on-6 configuration exhibited the lowest deformation under the present loading conditions, whereas the modeled All-on-5 configuration demonstrated the highest deformation (Figure 5). Increased deformation in the All-on-5 configuration may be due to reduced structural rigidity from uneven force distribution, whereas All-on-6 improves cross-arch stability.

### 3.5. Implant Deformation

**Figure 6:**
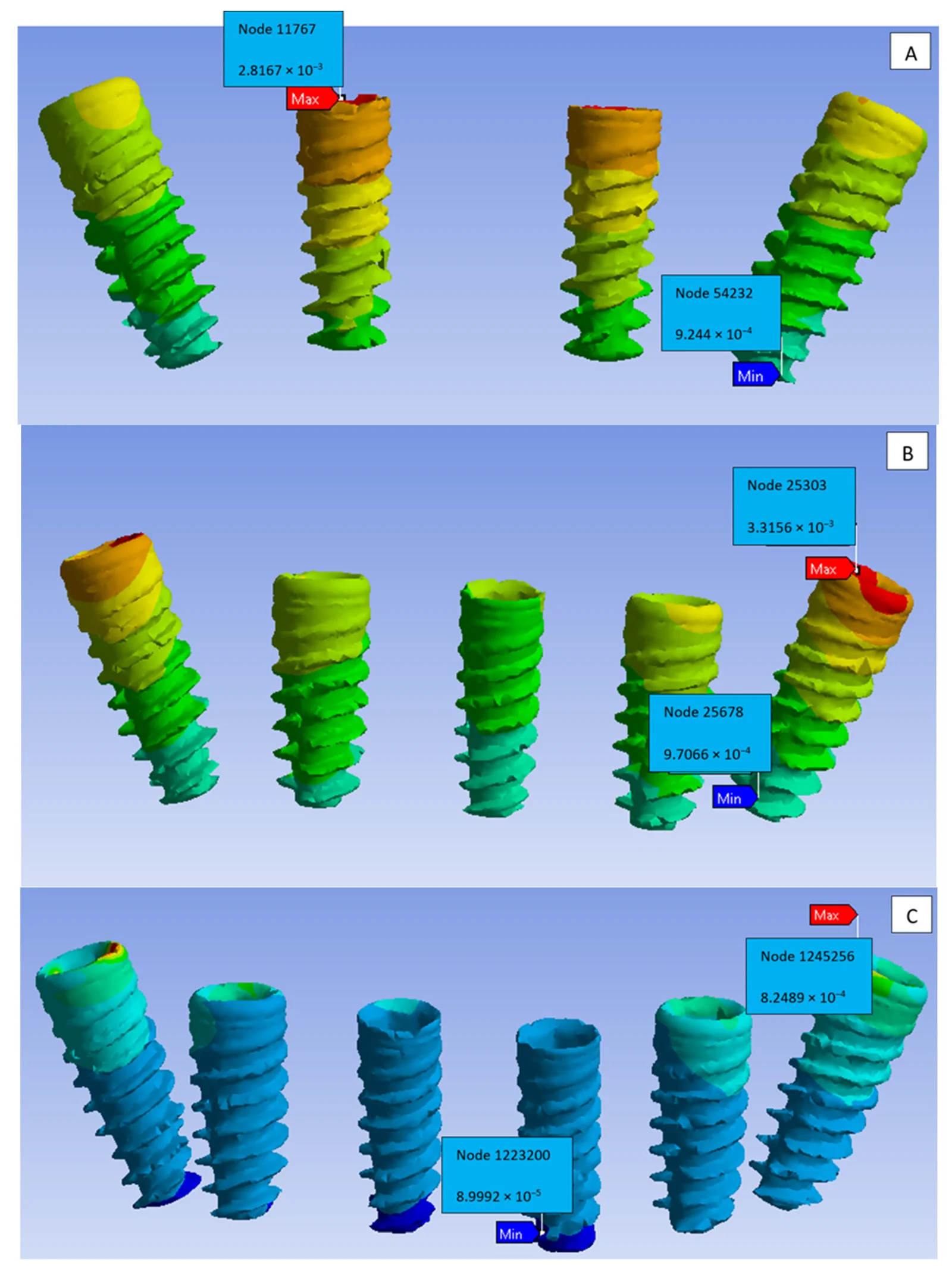
(**A**) Implant deformation in model 1; (**B**) implant deformation in model 2; (**C**) implant deformation in model 3.

Implant deformation followed a trend similar to total deformation. The lowest deformation was observed in Model 3 (8.2489 × 10^−4^ mm), followed by Model 1 (2.8167 × 10^−3^ mm) and Model 2 (3.156 × 10^−3^ mm). This indicates improved force dissipation and reduced micromovement at the implant level in the All-on-6 configuration (Figure 6).

### 3.6. Bone Deformation

**Figure 7:**
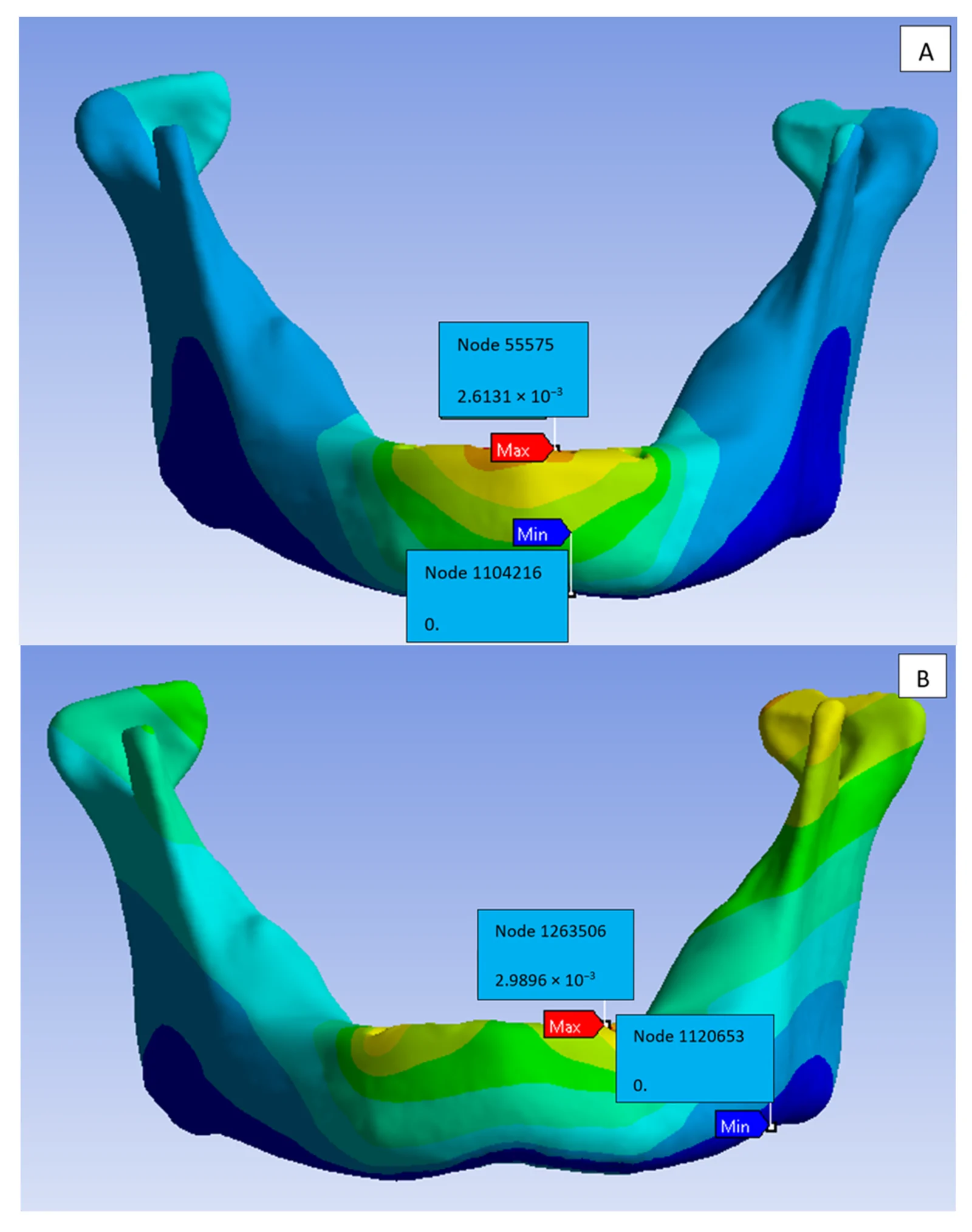

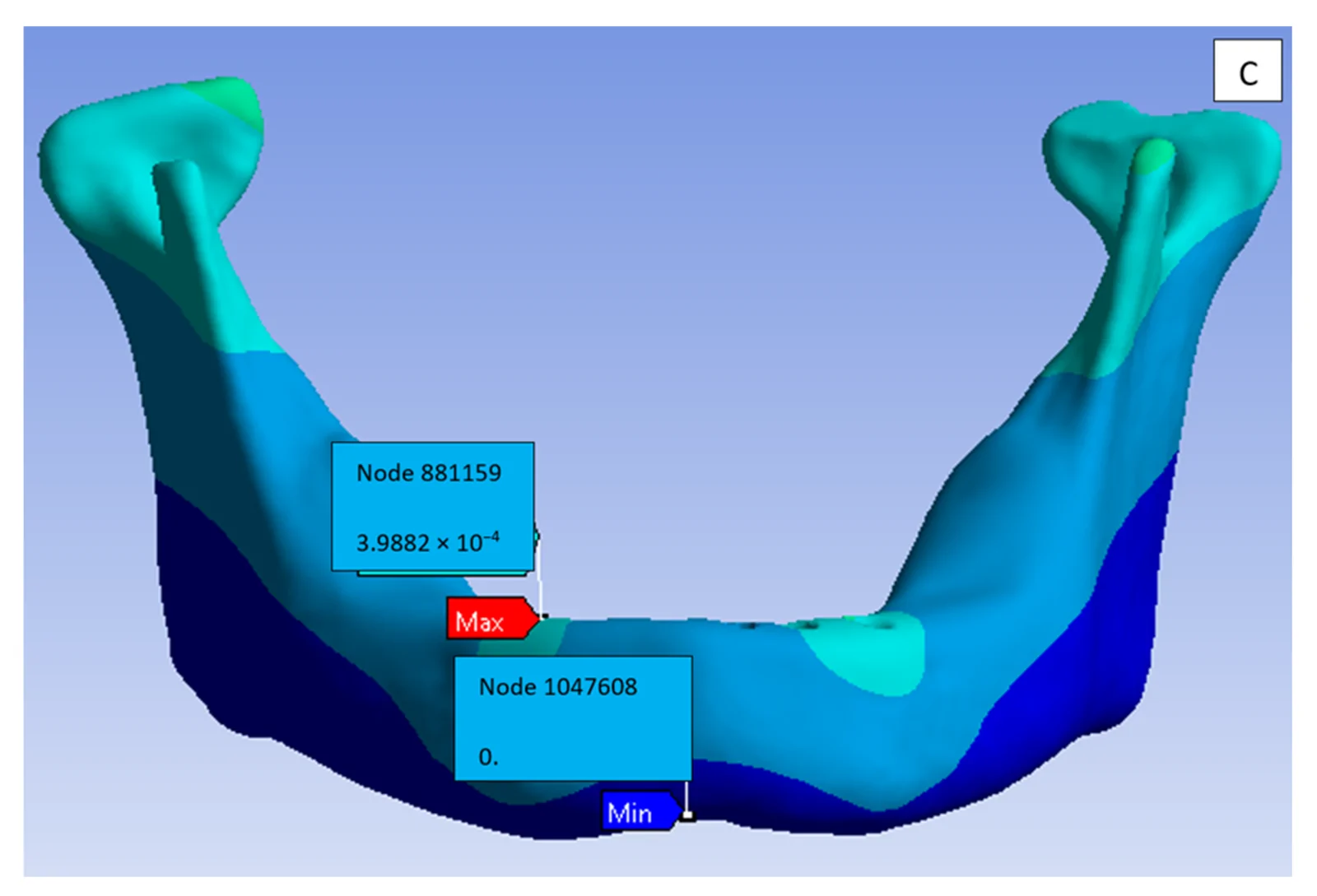
(**A**) Bone deformation in model 1; (**B**) bone deformation in model 2; (**C**) bone deformation in model 3.

Bone deformation values were 2.6131 × 10^−3^ mm for Model 1, increasing to 2.9896 × 10^−3^ mm in Model 2, and then markedly decreasing to 3.9882 × 10^−4^ mm in Model 3. This pattern reflects a progressive increase from Model 1 to Model 2, followed by a marked reduction in Model 3. These findings suggest that the All-on-6 configuration more effectively distributes occlusal forces, reducing both implant and bone strain, which is critical for long-term bone remodeling and biomechanical compatibility (Figure 7).

### 3.7. Comparative Biomechanical Pattern (Key Finding)

When all parameters are considered collectively, a distinct biomechanical pattern emerged (Table 3).

Overall, the All-on-4 configuration demonstrated the lowest bone stress but was associated with comparatively higher deformation. In contrast, the modeled All-on-5 configuration exhibited the highest stress and deformation among the three evaluated configurations under the present loading conditions. The observed biomechanical behaviour of the modeled All-on-5 configuration may be related to its specific implant distribution and load transfer characteristics. The All-on-6 configuration showed the lowest deformation and the most effective load distribution, suggesting superior overall biomechanical performance due to improved force sharing and reduced stress concentration.

## 4. Discussion

The present finite element analysis evaluated the biomechanical behaviour of three specific All-on-4, All-on-5, and All-on-6 implant configurations in an atrophic edentulous mandible. The findings demonstrate that implant number alone does not dictate biomechanical performance; rather, stress distribution and deformation are governed by a complex interplay of implant positioning, angulation, load transfer pathways, cantilever length, and prosthetic framework stiffness [21,22]. Because the evaluated models differed in both implant number and spatial distribution, the present findings represent a comparison of specific modelled configurations rather than implant number alone.

The overall von Mises stress analysis revealed a non-linear relationship with increasing implant number, with the All-on-5 configuration exhibiting the highest stress values, followed by All-on-6 and All-on-4. This finding challenges the conventional assumption that increasing implant number proportionally reduces biomechanical stress. Mechanistically, this behaviour can be attributed to non-uniform implant spacing and altered load transfer pathways in intermediate configurations. Suboptimal inter-implant distances may disrupt efficient load sharing, resulting in localized stress concentrations rather than uniform stress dissipation. Additionally, prosthetic framework stiffness and cantilever effects further influence force transmission across the arch, contributing to the observed non-monotonic stress patterns. Similar observations have been reported in recent finite element studies, emphasizing that implant positioning and prosthetic design exert a greater influence on stress distribution than implant number alone [23,24,25].

Analysis of implant-level stress further supported these findings, with higher stress concentrations observed in the All-on-5 configuration. In contrast, the All-on-6 model demonstrated improved load distribution, likely due to increased implant support facilitating more uniform force dissipation and reduced cantilever loading. Reduced stress concentration and improved load sharing are critical for minimizing micromotion at the implant–bone interface, which is essential for maintaining osseointegration and preventing mechanical complications [26].

Peri-implant bone analysis revealed that the All-on-4 configuration generated the lowest bone stress, whereas higher stress values were observed in the All-on-5 and All-on-6 models. However, deformation analysis demonstrated that the All-on-6 configuration exhibited the lowest total, implant, and bone deformation, indicating superior resistance to functional loading. These findings highlight the importance of evaluating both stress magnitude and deformation behaviour, as bone adaptation is primarily governed by strain distribution rather than stress alone [27]. From a mechanobiological perspective, configurations that promote more homogeneous strain distribution and reduced deformation may enhance long-term bone stability and minimize resorption [28].

The observed stress patterns are also influenced by implant geometry, dimensions, and in-bone positioning. Variations in implant length and diameter affect load transfer mechanisms and stress dissipation within the surrounding bone. Furthermore, bone geometry is not static and undergoes continuous remodeling during the healing and post-loading phases. Changes in bone density and architecture over time may alter biomechanical behaviour, potentially influencing long-term implant success. Therefore, the present findings should be interpreted as representing an initial mechanical scenario rather than a fully adaptive biological system [29].

Although von Mises stress was used in the present study for comparative evaluation, it should be recognized that bone behaves as an anisotropic and brittle material and does not strictly conform to von Mises failure criteria. Parameters such as maximum principal stress, principal strain and microstrain-based criteria may provide a more physiologically relevant assessment of bone response, as bone remodeling is regulated by strain distribution rather than stress magnitude alone. Additionally, stress homogeneity has been proposed as an important factor in optimizing load transfer and minimizing bone resorption. Therefore, while von Mises stress serves as a useful comparative metric, its limitations should be considered when interpreting biological relevance [30].

Although homogeneous, isotropic and linearly elastic material properties were assumed to simplify the finite element model, cortical and cancellous bone exhibit anisotropic and heterogeneous behaviour in vivo. In addition, bone density varies among patients and within different regions of the atrophic mandible, which may influence stress distribution and implant stability [29].

Although direct experimental validation was beyond the scope of this study, the observed stress distribution patterns are consistent with previous finite element studies of full-arch implant-supported prostheses. Similar investigations have shown that implant positioning and anterior–posterior spread influence stress transfer and framework deformation [27,28,29]. Nevertheless, further experimental and clinical validation is needed to confirm the predictive accuracy and clinical applicability of the present model.

The boundary conditions adopted in this study, particularly the constraint applied at the inferior border of the mandible and the exclusion of condylar regions, may influence the biomechanical response by limiting physiological mandibular flexure. In vivo, mandibular deformation under functional loading contributes to load redistribution across the arch. By restricting this deformation, the model may exhibit increased structural stiffness, potentially leading to higher localized stress concentrations around implants. Therefore, the present results may slightly overestimate stress magnitudes, and this limitation should be considered when extrapolating to clinical scenarios [31]. Similarly, the use of purely axial static loading represents a simplification of the complex biomechanical environment of the oral cavity. Axial loading was intentionally selected because it provides a standardized and reproducible loading condition, allowing direct comparison of the three implant configurations while minimizing confounding variables related to loading direction. In reality, occlusal forces are dynamic and multidirectional, incorporating oblique and lateral components as well as muscular forces. Previous studies have demonstrated that non-axial loading can significantly increase stress concentrations, particularly in cortical bone and at the implant neck, owing to increased bending movements [32]. Under such loading conditions, implant angulation, anterior–posterior spread, and framework rigidity may have a greater influence on load transfer, potentially altering the relative biomechanical performance of the evaluated configurations. Therefore, the ranking observed in the present study should be interpreted as applicable only to the axial static loading and boundary conditions employed, and future studies incorporating oblique, lateral, asymmetric and dynamic loading, together with physiological mandibular flexure, are warranted to determine whether similar biomechanical trends persist under more clinically realistic conditions.

Mandibular flexure represents another important biomechanical factor that was not incorporated into the present model. Physiological deformation of the mandible during functional movements can influence stress distribution in full-arch prostheses, particularly in rigidly splinted systems. Previous studies have demonstrated that mandibular flexure may affect prosthetic fit, stress concentration, and long-term implant success [33,34,35]. While the All-on-6 configuration demonstrated improved rigidity in the present study, the potential restriction of natural mandibular flexure should be considered in clinical scenarios.

From a biomechanical standpoint, the reduced deformation observed in the All-on-6 configuration suggests enhanced structural stability and resistance to bending forces. This may contribute to a lower risk of mechanical complications such as screw loosening, prosthetic fracture, and marginal bone loss, which are commonly associated with excessive deformation in implant-supported prostheses [36]. However, the findings also indicate that increasing implant number does not inherently improve biomechanical outcomes, as evidenced by the comparatively unfavorable performance of the All-on-5 configuration.

While the All-on-6 configuration demonstrated improved biomechanical stability under the present simulation conditions, these findings should not be interpreted as definitive clinical superiority. The results are derived from a simplified computational model that does not account for biological adaptation, bone remodeling, fatigue behavior, or patient-specific variability. Therefore, clinical decision-making should not be based solely on stress values but should integrate surgical, prosthetic, and biological considerations.

Within the specific finite element model evaluated in this study, the modeled All-on-5 configuration exhibited higher stress and deformation values than the modeled All-on-4 and All-on-6 configurations. This behavior appears to be influenced by the specific implant positioning, inter-implant spacing, and load transfer pathways adopted in the present simulation rather than implant number alone. Alternative All-on-5 implant distributions or prosthetic framework designs may produce different biomechanical outcomes. Therefore, these findings should be interpreted as configuration-specific rather than representative of the All-on-5 treatment concept as a whole. Nevertheless, the present results suggest that implant positioning and prosthetic design may have a greater influence on biomechanical performance than implant number alone, particularly in anatomically compromised conditions such as the atrophic mandible [37].

Clinically, the All-on-4 concept remains a well-established and cost-effective treatment modality, particularly in patients with limited bone availability [38]. In contrast, the All-on-6 configuration may provide enhanced biomechanical stability through improved load distribution and reduced deformation. Nevertheless, optimal treatment outcomes depend on comprehensive planning that considers implant positioning, angulation, prosthetic design, and patient-specific anatomical factors rather than implant number alone [39]. The All-on-4 configuration may be preferable in patients with limited bone volume or financial constraints, as it requires fewer implants and a less invasive surgical approach while still providing acceptable stress distribution. However, it may be associated with increased deformation due to reduced structural support [40].

The specific modelled All-on-5 configuration evaluated in the present study, despite the addition of an extra implant, did not demonstrate improved biomechanical performance and may represent a transitional design with suboptimal load distribution. The particular All-on-5 configuration modeled in this study showed less favorable biomechanical behavior under the tested conditions. These findings are specific to the modeled implant distribution and should not be generalized to the broader All-on-5 treatment concept. Within the limitations of the present finite element model, the modelled All-on-6 configuration demonstrated lower deformation and more favourable load distribution than the other evaluated configurations.

Although numerical differences in stress and deformation were observed among the three configurations, their clinical significance should be interpreted cautiously. The peak peri-implant bone stresses recorded in all models remained well below the reported yield strength of cortical bone, and the deformation values were extremely small (10^−3^ mm range). Therefore, these differences are unlikely to indicate immediate mechanical failure. However, whether such biomechanical differences translate into marginal bone loss, implant survival, or prosthetic complications cannot be determined from finite element analysis alone, as these outcomes are also influenced by biological remodeling, occlusion, and patient-specific factors.

The present simulations were based on a single mandibular anatomy, one implant distribution for each treatment concept, one prosthetic framework design, and simplified axial static loading conditions. Clinical treatment planning also depends on patient-specific anatomy, bone volume, interforaminal width, arch form, cantilever length, prosthetic material, occlusion, surgical complexity, biological response, and economic considerations. Therefore, the present results should be regarded as configuration-specific and hypothesis-generating rather than directly prescriptive for clinical implant selection.

## 5. Limitations

Despite providing valuable biomechanical insights into the All-on-4, All-on-5, and All-on-6 mandibular implant configurations, this study has several inherent limitations. The finite element model assumed homogeneous, isotropic, and linearly elastic material properties for bone and implant materials, whereas human bone is anisotropic and heterogeneous. The applied boundary conditions, including fixation at the inferior mandibular border and exclusion of condylar regions, may restrict physiological mandibular flexure and introduce a degree of artificial stiffness, potentially leading to overestimation of peri-implant stresses. The analysis was performed under static loading conditions and only axial forces were considered, while actual masticatory forces are dynamic and multidirectional. This simplification may influence the magnitude and distribution of stresses observed. Soft tissues, including the peri-implant mucosa and periodontal ligament, were not modeled, although they may influence load transmission. In addition, the mandibular flexure phenomenon, which may affect stress transmission to implants in splinted full-arch restorations, was not reflected in the present finite element model. Long-term biological responses such as bone remodeling, changes in osseointegration, and implant fatigue were also not considered. Therefore, the findings should be interpreted as comparative biomechanical trends rather than definitive clinical outcomes. Additionally, the results are model-specific and may not be directly extrapolated to all clinical implant configurations.

## 6. Future Perspective

Future studies should incorporate patient-specific anatomy, bone quality, and dynamic masticatory loading to better simulate clinical conditions. Modeling soft tissues and long-term biological responses, such as bone remodeling and implant fatigue, would improve predictive accuracy. Clinical validation of FEA findings and advanced prosthetic designs can guide optimal implant number and placement for full-arch mandibular prostheses.

## 7. Conclusions

Finite element analysis demonstrated that implant configuration influences stress distribution and deformation in full-arch mandibular prostheses. Within the specific modelled configurations evaluated, the All-on-5 configuration exhibited higher stress concentrations and deformation under the present simulation condition, indicating less favorable biomechanical behavior than the modelled All-on-4 and All-on-6 configurations. The modeled All-on-4 configuration showed lower bone stress, whereas the modeled All-on-6 configuration demonstrated lowest deformation among the three evaluated configurations under the applied loading conditions.

The findings indicate that biomechanical performance depends not only on implant number but also on implant spatial distribution and load transfer characteristics. Accordingly, the present study compares three specific implant configurations representing the All-on-4, All-on-5 and All-on-6 treatment concepts rather than implant number as an isolated variable.

Within the limitations of this finite element study, the modeled All-on-6 configuration demonstrated more favorable biomechanical behavior under the applied loading conditions, while the modeled All-on-5 configuration exhibited comparatively higher stress concentrations. However, these findings are configuration-specific and depend on the assumed geometry, implant positioning, prosthetic framework and loading conditions. Therefore, they should be regarded as hypothesis-generating rather than directly prescriptive for clinical implant selection. Clinical decision-making should also consider biological factors, patient-specific anatomy, prosthetic design, occlusion and long-term functional outcomes.

## Figures and Tables

**Table 1:** Young’s modulus and Poisson ratio.

Material	Young’s Modulus (MPa)	Poisson’s Ratio	Tensile Yield Strength (MPa)
Titanium	110,000	0.35	834
Cortical bone	13,700	0.3	114
Cancellous bone	1370	0.3	52

**Table 2:** Stress and deformation in model 1, model 2 and model 3.

Parameter	Model 1 (Min)	Model 1 (Max)	Model 2 (Min)	Model 2 (Max)	Model 3 (Min)	Model 3 (Max)
Overall Von Mises Stress (MPa)	1.3882 × 10^−9^	38.016	6.0168 × 10^−9^	76.821	1.3774 × 10^−8^	67.996
Von Mises Stress in Implants (MPa)	0.20455	38.016	0.13169	76.821	0.009688	67.996
Von Mises Stress in Bone (MPa)	1.3882 × 10^−9^	5.787	6.0168 × 10^−9^	16.443	1.3774 × 10^−8^	13.486
Total Deformation (mm)	0	2.8167 × $10^{-3}$	0	3.156 × 10^−3^	0	8.2489 × 10^−4^
Implant Deformation (mm)	9.244 × 10^−4^	2.8167 × 10^−3^	9.7066 × 10^−4^	3.156 × 10^−3^	8.9992 × $10^{-5}$	8.2489 × 10^−4^
Bone Deformation (mm)	0	2.6131 × 10^−3^	0	2.9896 × 10^−3^	0	3.9882 × 10^−4^

**Table 3:** Biomechanical performance comparison of implant-supported configurations.

Parameter	Best Model	Worst Model
Overall Stress	All-on-4	All-on-5
Bone Stress	All-on-4	All-on-5
Deformation	All-on-6	All-on-5
Structural Stability	All-on-6	All-on-5

## Data Availability

The original contributions presented in this study are included in the article/Supplementary Materials. Further inquiries can be directed to the corresponding author.

## Supplementary Materials

The following supporting information can be downloaded at: https://www.mdpi.com/article/10.3390/dj14080480/s1, Figure S1: Finite element mesh generated for the three-dimensional mandibular model showing the tetrahedral mesh and mesh statistics (nodes and elements) used for finite element analysis; Figure S2: ANSYS mesh settings illustrating the inflation and smoothing parameters applied during finite element mesh generation of the mandibular model; Figure S3: ANSYS mesh quality assessment of the mandibular finite element model showing mesh quality metrics used to evaluate the generated tetrahedral mesh; Figure S4: ANSYS mesh configuration showing element sizing and curvature-based meshing parameters used to generate the finite element mesh of the mandibular model.
