# Supplementary material for: Biomechanical Comparative Evaluation of the Atrophic Edentulous Mandible Using All-on-4, All-on-5 and All-on-6 Concepts: A Finite Element Analysis
Source: Dent J (Basel). 2026 Aug 5;14(8):480. doi: 10.3390/dj14080480 (PMC13511435; doi:10.3390/dj14080480)
Supplement: Supplementary file 1 [file dentistry-14-00480-s001.zip › dentistry-4382061-supplementary.pdf]

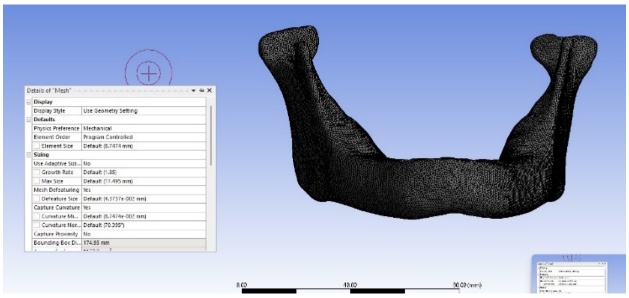

Figure S1- Finite element mesh generated for the three-dimensional mandibular model showing the tetrahedral mesh and mesh statistics (nodes and elements) used for finite element analysis

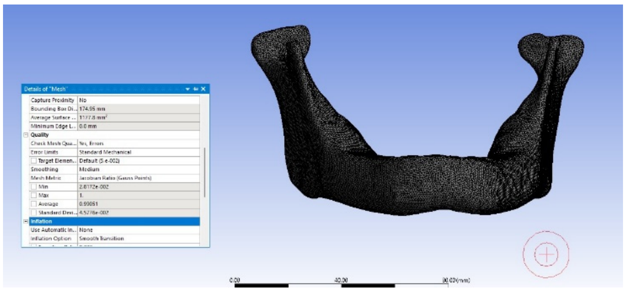

Figure S2- ANSYS mesh settings illustrating the inflation and smoothing parameters applied during finite element mesh generation of the mandibular model

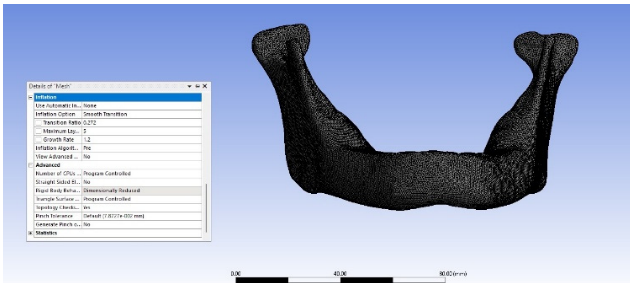

Figure S3- ANSYS mesh quality assessment of the mandibular finite element model showing mesh quality metrics used to evaluate the generated tetrahedral mesh

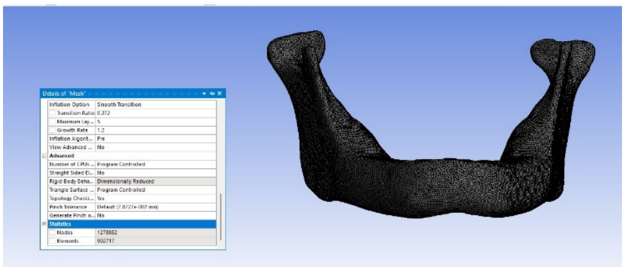

Figure S4- ANSYS mesh configuration showing element sizing and curvature-based meshing parameters used to generate the finite element mesh of the mandibular model
